# Deregulation of SET is Associated with Tumor Progression and Predicts Adverse Outcome in Patients with Early-Stage Colorectal Cancer

**DOI:** 10.3390/jcm8030346

**Published:** 2019-03-12

**Authors:** Ion Cristóbal, Blanca Torrejón, Jaime Rubio, Andrea Santos, Manuel Pedregal, Cristina Caramés, Sandra Zazo, Melani Luque, Marta Sanz-Alvarez, Juan Madoz-Gúrpide, Federico Rojo, Jesús García-Foncillas

**Affiliations:** 1 Cancer Unit for Research on Novel Therapeutic Targets, Oncohealth Institute, IIS-Fundación Jiménez Díaz-UAM, E-28040 Madrid, Spain; blanca.torrejon@quironsalud.es (B.T.); jaime.rubiop@quironsalud.es (J.R.); manuel.pedregal@quironsalud.es (M.P.); ccarames@fjd.es (C.C.); 2 Translational Oncology Division, Oncohealth Institute, IIS- Fundación Jiménez Díaz-UAM, E-28040 Madrid, Spain; andreasantos.asc@gmail.com; 3 Medical Oncology Department, University Hospital “Fundación Jiménez Díaz”, UAM, E-28040 Madrid, Spain; 4 Pathology Department, IIS-Fundación Jiménez Díaz-UAM, E-28040 Madrid, Spain; szazo@fjd.es (S.Z.); melani.luque@quironsalud.es (M.L.); marta.sanza@quironsalud.es (M.S.-A.); jmadoz@fjd.es (J.M.-G.)

**Keywords:** SET, protein phosphatase 2A (PP2A), early-stage colorectal cancer (CRC)

## Abstract

SET nuclear proto-oncogene (SET) deregulation is a novel molecular target in metastatic colorectal cancer (CRC). However, its role in CRC progression and its potential clinical impact in early-stage CRC patients remain unknown. Here, we studied the biological effects of SET on migration using wound-healing and transwell assays, and anchorage-independent cell growth using soft agar colony formation assays after ectopic SET modulation. SET was analyzed by immuno-staining in 231 early-stage CRC patients, and miR-199b expression was quantified by real-time PCR in a set of CRC patients. Interestingly, SET enhances cell migration, markedly affects the colony-forming ability, promotes epithelial to mesenchymal transition, and induces the expression of the MYC proto-oncogene (c-MYC) in CRC cells. SET overexpression was detected in 15.4% of cases and was associated with worse Eastern Cooperative Oncology Group (ECOG) status (*p* = 0.021) and relapse in stage-II CRC patients (*p* = 0.008). Moreover, SET overexpression predicted shorter overall survival (*p* < 0.001) and time to metastasis (*p* < 0.001), and its prognostic value was particularly evident in elderly patients. MiR-199b downregulation was identified as a molecular mechanism to deregulate SET in patients with localized disease. In conclusion, SET overexpression is a common alteration in early-stage CRC, playing an oncogenic role associated with progression and aggressiveness, and portends a poor outcome. Thus, SET emerges as a novel potential molecular target with clinical impact in early-stage in CRC.

## 1. Introduction

Colorectal cancer (CRC) has the highest incidence rate of all gastrointestinal cancers and is the third most commonly diagnosed of all cancer types and the fourth highest cause of cancer-related deaths worldwide [1]. Disease stage at diagnosis remains the single most critical factor determining the patient outcome [2]. Though patients with metastatic disease have the worst prognosis, more than 70% of newly diagnosed cases have surgically resectable, localized disease [3,4,5]. Therefore, further improvements in our knowledge of the molecular mechanisms that govern CRC progression are necessary to prevent metastasis. In particular, identification of novel biomarkers and the development of alternative therapeutic strategies are required to impair disease progression.

The protein phosphatase 2A (PP2A) is a tumor suppressor that is frequently inactivated in human cancer due to its pivotal role in controlling the activation of critical signaling pathways for the tumor cell [6,7]. Several published works have reported that functional inactivation of PP2A is common and has great importance in CRC [8,9,10,11], and that overexpression of the endogenous PP2A inhibitor SET [12] is a key molecular mechanism to inhibit PP2A in this disease [13]. SET regulates a wide variety of molecular processes in the cell such as histone acetylation and transcription, migration, stemness, and cell-cycle regulation [14,15,16,17,18]. In addition, the oncoprotein SET modulates the activation status of the transcription factor activator protein-1 (AP-1) deregulates protein kinase B (AKT) signaling, inhibits the DNase activity of NM23-H1, and negatively regulates p53 activity [19,20,21,22].

Furthermore, SET has been found to be de-regulated in some solid tumors and leukemias [23,24,25,26,27,28,29], and it has been proposed as a novel target for anticancer therapy [30,31,32,33,34,35]. Of importance, some data in the literature indicate that SET could also be playing a relevant role in CRC through the regulation of the Wingless-type MMTV integration site family (WNT) signaling pathway [36]. Our group reported that SET deregulation determines poor outcome in metastatic CRC patients by promoting cell growth and decreasing sensitivity to standard chemotherapeutic agents such as oxaliplatin and 5-fluorouracil [12]. Regarding the molecular mechanisms responsible for SET deregulation, the transcription factor ecotropic virus integration site-1 (EVI-1) has been reported to promote SET expression in acute myeloid leukemia, and miR-199b has been found to be negatively regulate SET in choriocarcinoma [24,37]. Interestingly, SET was identified as a direct target of miR-199b, and the downregulation of this microRNA was described as the molecular mechanism responsible for SET overexpression in around half of metastatic CRC patients with high SET levels [38].

In this study, we further explore the role of SET in CRC progression and evaluate, for the first time, its potential clinical impact in CRC patients with localized disease. We observed that SET promotes cell migration, colony-forming ability, and epithelial to mesenchymal transition (EMT) in CRC cells. To determine its clinical relevance, we quantified SET in a series of 231 CRC patients without metastatic disease at diagnosis, observing that SET overexpression is a common alteration that is associated with relapse and predicts shorter overall survival and time to metastasis. Moreover, we identified miR-199b downregulation as a contributing molecular mechanism to deregulate SET in early-stage CRC patients.

## 2. Experimental Section

### 2.1. Cell Cultures and Transfection

The human CRC cell lines SW480 (ATCC CCL-228) and HT-29 (ATCC HTB-38) were purchased from American Type Culture Collection (ATCC, Manassas, VA, USA). Authentication was done by the authors in all cases (LGC Standards, Wesel, Germany). Cell lines were maintained in RPMI-1640 (Invitrogen, Carlsbad, CA, USA) with 10% fetal bovine serum (FBS) and were grown at 37 °C in a 5% CO_2_ atmosphere. Media were supplemented with penicillin G (100 U/mL), and streptomycin (0.1 mg/mL). For transfection experiments, CRC cells were seeded in 6-well plates and transfected with 10 μL of Lipofectamine 2000 (Invitrogen, Carlsbad, CA, USA) and 2 μg of plasmidic vectors or 75 nM of SET-specific siRNAs designed and synthesized by Dharmacon RNA Technologies (Dharmacon, Lafayette, CO, USA).

### 2.2. Patient Samples

Primary colorectal tissues were surgical resection specimens from CRC tumors obtained from Fundacion Jimenez Diaz Biobank (BFJD, Madrid, Spain). The study comprised consecutive formalin-fixed paraffin-embedded (FFPE) tumor samples from 247 CRC patients without metastatic CRC, retrospectively selected from 2001 to 2012 according to the following criteria: Adenocarcinoma, operable disease, no neo-adjuvant therapy, sufficient available tissue, available clinical follow-up data, and metastatic disease. Tumor, Node, Metastases (TNM) staging was performed using the 7th American Joint Committee on Cancer (AJCC) staging system for CRC. Clinical data were collected from medical records by oncologists (MP, CC, and JR). KRAS proto-oncogene, GTPase (KRAS) mutational status was determined by Cobas KRAS Mutation Test kit (Roche Molecular Diagnostics, Pleasanton, CA, USA) following the manufacturer’s procedures. Tissue microarrays (TMA) were constructed. Representative areas of each tumor were carefully selected and three tissue cores (1 mm diameter) were obtained using a TMA workstation (T1000 Chemicon). Samples were taken anonymously. The ethical committee and institutional review board approved the project.

### 2.3. Wound-Healing Assay

A total of 8 × 10^5^ cells per well were seeded in 6-well plates and allowed to adhere for 24 h in complete medium. The monolayer was artificially injured by scratching across the plate with a 10 μL pipette tip. Wells were then washed twice with phosphate-buffered saline (PBS) to remove detached cells and wound healing was monitored using a Leica DMi1 (Leica, Wetzlar, Germany) microscope and the image acquisition software Leica Application Suite version 4.5. Images were captured at the beginning and at regular intervals during cell migration to close the wound. Comparisons were performed to quantify the migration rate of the cells between the different experimental conditions. Relative cell migration are represented in the histograms considering the percentage of healed area after ectopic SET silencing or overexpression and compared to control conditions.

### 2.4. Transwell Migration Assay

Migration assays were performed using 24-well plates with transwell permeable supports of 6.5 mm insert and a polycarbonate membrane with an 8-µm pore size (Costar 3422, Corning Inc., Corning, NY, USA). Cells were seeded in the upper chamber at 2 × 10^4^ cells/mL in 0.1 mL of serum-free RPMI-1640 media. A volume of 0.8 mL of media supplemented with 10% FBS was placed in the bottom well as a chemo-attractant. After incubation for 24 h at 37 °C in an atmosphere containing 5% CO_2_, migrated cells on the lower surface were stained using crystal violet and counted under a light microscope.

### 2.5. Colony-Forming Assay

Experiments were performed in 6-well plates coated with 3 mL of 0.6% soft agarose (Sigma, St. Louis, MO, USA) in medium. A total of 5 × 10^3^ cells were suspended in 0.3% agarose in medium and plated in triplicates over the pre-coated wells. Fresh medium was supplied twice a week. After 10 days, colonies were stained with MTT (M-565, Sigma, St. Louis, MO, USA) for 4 h at 37 °C. Then, colonies were fixed by adding dimethyl sulfoxide (DMSO) overnight at 37 °C. Colony numbers were determined from triplicates and three independent experiments were carried out for each condition and cell line.

### 2.6. Western Blot Analysis

Protein extracts were isolated using TRIzol Reagent (Invitrogen) following manufacturer’s indications, clarified (12,000× g, 15 min, 4 °C), denatured and subjected to SDS-PAGE and Western-blot. Antibodies used were rabbit polyclonal anti-SET (Santa Cruz Biotechnology, Dallas, TX, USA), rabbit polyclonal anti-E-cadherin (24E10), anti-N-cadherin (D4R1H) and anti-Vimentin (D21H3) (Cell Signaling Technology, Leide, The Netherlands), rabbit polyclonal anti-c-myc (Ab32072) (Abcam, Cambridge, UK), and mouse monoclonal anti-βactin (Sigma, St. Louis, MO, USA). Proteins were detected with the appropriate secondary antibodies conjugated to alkaline phospatase (Sigma, St. Louis, MO, USA) by chemiluminescence using Tropix CSPD and Tropix Nitro Block II (Applied Biosystems, Foster City, CA, USA).

### 2.7. Immunohistochemistry

Tissue sections (3 μm) were mounted on positively charged glass slides. After de-paraffinization in xylene and graded alcohols, heat antigen retrieval was performed in EDTA-based buffer, pH9 (Dako). Endogenous peroxidase was blocked by 0.03% hydrogen peroxide for 5 min. Slides were incubated with the same primary antibody used against SET as described above, for 60 min at room temperature, followed by appropriate anti-Ig horseradish peroxidase-conjugated polymer (Flex+, Dako). Sections were visualized with 3,3′-diaminobenzidine as a chromogen. All staining’s were performed in a Dako Autostainer. Sections incubated with normal non-immunized rabbit immunoglobulins were used as negative controls. As a positive control, a section of colorectal tumor with known expression of SET was used. SET antibody sensitivity (1:5000) had been calculated in a range of crescent dilutions of the primary antibody. Specificity was confirmed in a set of paired fresh frozen and FFPE samples were processed by Western blot and immunohistochemistry (IHC). Only the membrane of epithelial cells, but not stromal cells, was evaluated for SET expression by two pathologists blinded to clinical data (FR and SZ). A semi-quantitative histoscore was calculated by estimating the percentage of tumor cells positively stained with low, medium, or high staining intensity. The final score was determined after applying a weighting factor to each estimate. The following formula was used: Histoscore = (low %) × 1 + (medium %) × 2 + (high %) × 3 and the results ranged from 0 to 300.

### 2.8. Statistical Analysis

Statistical analyses were performed using SPSS 20 for windows (SPSS Inc, Chicago, IL USA). Overall survival (OS) was defined as the time from the date of surgery to the date of death from any cause or last follow-up. Time to metastasis (TTM) was defined as the time from surgery until distant recurrence in those cases with metachronous metastases. Kaplan-Meier plots and survival comparisons were performed by means of log-rank test if the proportional hazard assumption was fulfilled and Breslow otherwise. The Cox proportional hazards model was adjusted taking into consideration significant parameters in the univariate analysis. The cutoff point for SET expression was determined as previously described for metastatic CRC [12]. This work was carried out in accordance with Reporting Recommendations for Tumor Marker Prognostic Studies (REMARK) guidelines [39]. Data represented for transfection experiments are mean of three independent experiments ± s.d. Statistical comparisons were carried out by 2-sided *t*-test analyses. A *p*-value less than 0.05 was considered statistically significant.

### 2.9. Quantification of miRNA Expression Levels

Total RNA was isolated using RecoverAll Total Nucleic Acid Isolation kit (Thermo Fisher, Waltham, MA, USA) according to manufacturer’s instructions. Samples were reverse transcribed using the TaqManHMicroRNA Reverse Transcription Kit (Applied Biosystems, Foster City, CA, USA) and mature miRNAs were quantified by quantitative real-time RT-PCR using TaqMan MicroRNA Assays (Applied Biosystems, Foster City, CA, USA), specific for miR-199b and U6B as an internal control. Analysis of relative gene expression data was performed using the 2^−ΔΔ*C*_T_^ method [40], where ∆∆*C*_T_ = (*C*_T,Target Gene_ − *C*_T,U6B_)_Cell Line_ − (*C*_T,Target Gene_ − *C*_T,U6B_)_Normal Control_.

## 3. Results

### 3.1. SET Plays a Relevant Role in Regulating Cell Migration of CRC cells

**Figure 1:**
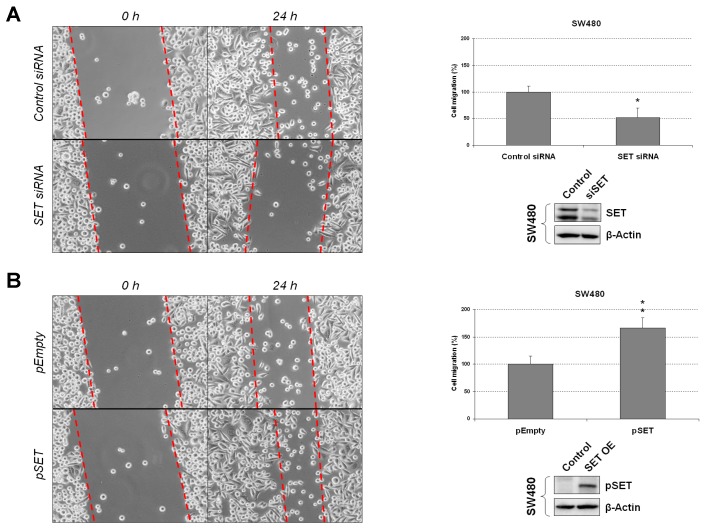
SET modulates cell migration in colorectal cancer (CRC) cells. Wound-healing assay showing migration in SW480 cells after ectopic (**A**) SET silencing or (**B**) SET overexpression. Dashed lines represent the migration border; OE: Overexpression; * *p* < 0.05; ** *p* < 0.01.

To investigate the biological significance of SET in CRC progression, we first assessed the effects of an ectopic SET modulation on cell migration. Interestingly, we observed significantly decreased migration in SW480 cells after SET silencing in comparison with those cells transfected with a negative control siRNA (Figure 1A). Similar results were obtained with the HT-29 cell line (Figure S1A). In concordance with these results, SET overexpression in both SW480 and HT-29 cells enhanced cell migration compared to cells transfected with an empty vector (Figure 1B and Figure S1B). In order to exclude a possible functional effect on off-targets, we performed a rescue experiment with a SET expression vector, as well as a time-course analysis in both SW480 (Figure S2) and HT-29 cells (Figure S3), observing that ectopic SET expression restored migration capability in both cell lines after SET silencing.

**Figure 2:**
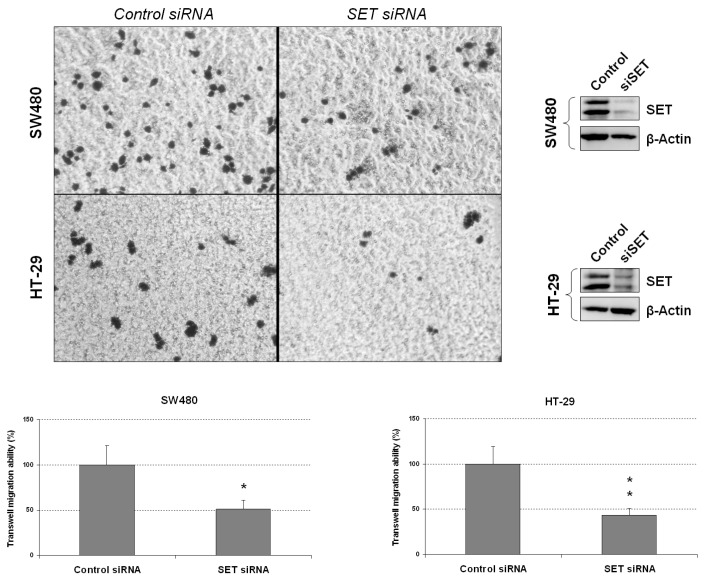
SET silencing inhibits transwell migration in CRC cells. Transwell migration assay in SW480 and HT-29 cells after SET silencing; * *p* < 0.05; ** *p* < 0.01.

To further confirm the role of SET in modulating cell migration in CRC cells, we carried out a transwell migration assay using SW480 and HT29 cells. Interestingly, SET silencing dramatically decreased transwell migration in both cell lines compared to negative control cells (Figure 2), thereby evidencing that SET deregulation plays a relevant role in regulating the migration of CRC cells. In order to exclude a possible functional effect on off-targets we performed a rescue experiment of transwell with a SET expression vector. As expected, we observed that ectopic expression of SET restored cell migration to levels similar to control conditions in both SW480 and HT-29 cells (Figure S4).

### 3.2. Deregulation of SET Markedly Affects Colony-Forming Ability and Regulates EMT of CRC Cells

**Figure 3:**
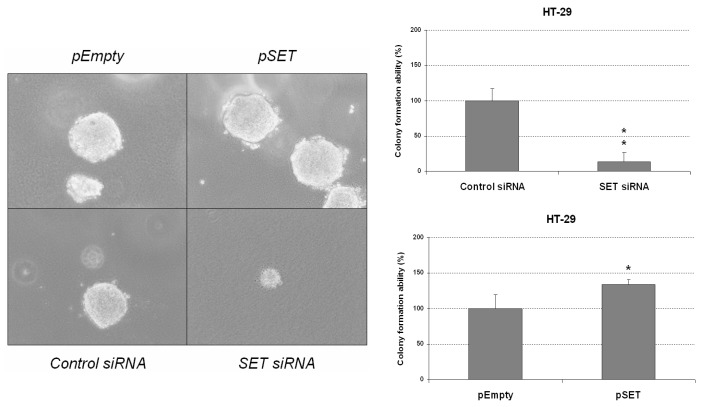
SET deregulation affects CRC colony-forming ability. Colony-forming assays showing the effect of SET silencing and SET overexpression on the anchorage-independent cell growth of HT-29 cells; * *p* < 0.05; ** *p* < 0.01.

In order to further explore the potential significance of SET in CRC progression and aggressiveness, we next performed colony-formation assays in soft agar to analyze whether SET deregulation can alter the malignancy of CRC cells measured as anchorage-independent cell growth. We observed that colony formation was markedly impaired in HT-29 cells after SET silencing; conversely, colony-forming ability was found to be significantly enhanced in HT-29 cells ectopically expressing SET compared to normal controls (Figure 3). While these experiments were also performed in SW480 cells, this cell line failed to form colonies in any of the conditions tested.

Furthermore, we analyzed the potential role of SET regulating EMT and proteins involved in CRC progression and metastasis such as c-MYC. Interestingly, we found that SET silencing resulted in higher E-cadherin levels concomitant with a decrease in the expression of proteins of a mesenchymal phenotype such as N-cadherin and vimentin in SW480 and HT-29 cells. In concordance with these results, ectopic SET expression decreased E-cadherin levels and increased N-cadherin and vimentin expression (Figure S5). Moreover, we also analyzed the expression levels of c-MYC, an oncoprotein largely involved in progression to metastatic disease since it positively regulates EMT during carcinogenesis [41]. Interestingly, we observed that SET positively regulates c-MYC expression in both SW480 and HT-29 cell lines (Figure S5). Altogether, these results appear to indicate that SET is involved in CRC aggressiveness by promoting colony-forming ability and EMT of CRC cells.

### 3.3. Prevalence of SET Overexpression in Early-Stage CRC and Its Association with Molecular and Clinical Parameters

To study the prevalence and clinical significance of SET overexpression, we quantified the expression of SET by immunohistochemistry in a cohort of 247 CRC patients without metastatic disease at diagnosis. Patient characteristics are presented in Table S1, and immuno-histochemical detection of SET is shown in Figure S6A. Interestingly, SET overexpression was found in 34 of 231 cases (15.4%). We found this alteration to be associated with worse ECOG performance status (33.3% vs. 13.2%, *p* = 0.021). As ECOG status is influenced by tumor stage and age, we also analyzed the association of ECOG status with these parameters, observing a correlation between high ECOG and stage III (*p* = 0.014). Moreover, these patients are mainly older, but in this case, statistical significance was not achieved (*p* = 0.060), probably due to the low number of cases with high ECOG (Table S2). While the prevalence of SET overexpression was higher in patients with stage III than in those cases with stage I-II disease, the differences did not reach statistical significance in this case (17.7 % vs. 11%, *p* = 0.156). The association between SET overexpression and molecular and clinical parameters are included in Table 1.

We also analyzed the potential association of SET overexpression with relapse (local or distant), though our findings were not statistically significant. We next stratified our cohort by stage; to our surprise, we found that SET overexpression strongly associates with relapse in those CRC patients with stage II at diagnosis (*p* = 0.008), whereas significance was far from being achieved in the subgroup of CRC patients with stage III disease (*p* = 0.882) (Table 2).

### 3.4. Clinical Significance of SET Overexpression in Early-Stage Colorectal Cancer

**Figure 4:**
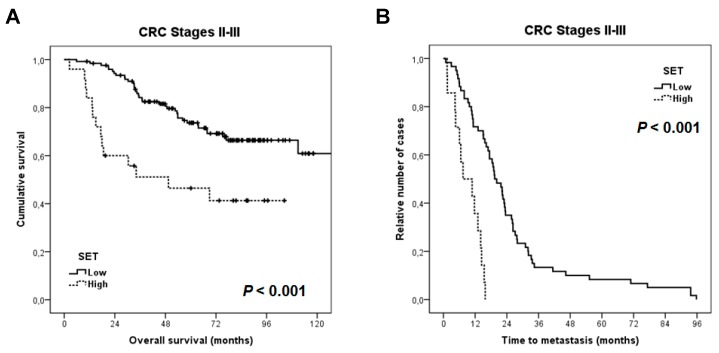
Kaplan-Meier analyses for SET expression in early-stage CRC patients: (**A**) Overall survival; and (**B**) time to metastasis (*n* = 87).

For survival analyses, we included 149 cases with stages II and III for whom clinical follow-up data were available, 89 were male and 60 female, with a median of age and OS of 72 years (range: 36–93) and 66.4 months, respectively. Patients with stage I CRC were excluded due to the limited number of cases with clinical data available. Interestingly, we found that the subgroup of patients in whom SET was overexpressed showed a substantially shorter OS (57 vs. 113.2 months, *p* < 0.001) (Figure 4A). We observed that in the subgroup of cases that developed metachronous metastases (*n* = 87), SET overexpression predicted a significantly shorter time to metastasis (*p* < 0.001) (Figure 4B). As in previous reported data showing that SET overexpression predicts poor outcome in metastatic CRC, this alteration was also predictive of shorter time to progression from metastatic diagnosis (*p* = 0.001) (Figure S6B). Moreover, multivariate analysis demonstrated that SET overexpression is an independent predictor of poor outcome in our cohort (Table 3).

We next analyzed the clinical impact of SET overexpression after stratifying our cohort by age. Interestingly, we observed that the prognostic impact of SET was significant in the subgroup of elderly patients (45.4 vs. 103.9 months, *p* < 0.001), whereas significance was not achieved in the subgroup of younger patients (45.4 vs. 103.9 months, *p* < 0.001) (Figure S6C). Of note, multivariate analysis demonstrated that SET overexpression was an unfavorable independent factor associated with OS in the subgroup of elderly patients (Table S3).

### 3.5. MiR-199b Downregulation is a Contributing Alteration to Deregulate SET in Localized Colorectal Cancer

Previous data reporting that miR-199b directly targets SET in CRC and that miR-199b downregulation is a frequent event that facilitates SET overexpression in metastatic CRC prompted us to analyze the potential contribution of this alteration to deregulate SET also in early-stage CRC. Thus, we analyzed the expression levels of miR-199b in 29 patients from our cohort with sufficient available material. Patient characteristics are presented in Table S4. We observed SET overexpression in seven cases and miR-199b downregulation in four out of 29 cases. Interestingly, we found that miR-199b downregulation was significantly associated with SET overexpression (*p* = 0.010) (Table S5). To further investigate the potential role of miR-199b in regulating SET, we next compared the miR-199b expression profiles between the subgroups of SET-overexpressing and non-overexpressing patients. In a noteworthy finding, we observed that miR-199b was significantly downregulated in the subgroup of cases with high SET expression (*p* = 0.004) (Figure S7). Therefore, miR-199b downregulation is a molecular mechanism that contributes to SET deregulation in CRC patients without metastatic disease at diagnosis.

## 4. Discussion

The upregulation of the SET oncogene has been has been described in several tumor models, and over time it has emerged as a promising molecular target for alternative therapeutic strategies in these cancers [23,24,25,26,27,28,29,30,31,32,33,34,35]. Our previous findings showed that PP2A inhibition is a frequent event in CRC, and we identified SET deregulation as a key mechanism to inhibit PP2A [8]. Moreover, we observed that SET promotes cell proliferation and colonosphere formation, impairing PP2A antitumor activities and modulating the sensitivity of CRC cells to oxaliplatin treatment. Clinically, we showed that SET overexpression is a recurrent alteration that predicts adverse outcome and induces decreased sensitivity to oxaliplatin in metastatic CRC. However, its potential significance in CRC progression and in early-disease stages remains to be investigated. Altogether, existing data led us to hypothesize that SET could also play an important oncogenic role in CRC progression, regulating both migration and colony-forming capabilities, as well as having clinical impact in the early stages of the disease.

In order to study the functional relevance of SET deregulation in CRC progression, we ectopically modulated SET in two different CRC cell lines and observed that SET silencing induced a decrease in cell migration and anchorage-independent cell growth in both cell lines. In contrast, SET-overexpressing CRC cells showed increased migration, colony-forming abilities, and EMT and c-MYC levels (Figure 1, Figure 2 and Figure 3 and Figures S1–S5). These results indicate that SET deregulation plays a potential oncogenic role in CRC progression.

To further investigate the significance of SET deregulation in CRC, we evaluated its expression in earlier stages of CRC, studying a cohort of 231 CRC patients without metastatic disease. Interestingly, SET overexpression had a lower prevalence in this cohort (15.4%) (Table 1) compared to that previously reported for metastatic CRC (24.8%) [12]. In agreement with previous data on metastatic CRC, SET overexpression was found to be associated with worse ECOG performance. However, this alteration was significantly associated with those metastatic CRC cases with colon tumors (31.8% vs. 11.8%), whereas, in the present study, we observed even higher frequency in the subgroup of patients with rectal tumors (15.2% vs. 17.1%). Therefore, the prevalence of this alteration does not undergo significant changes in rectal cancer, but seems to progressively enhance its prevalence during the disease progression in patients with colon tumors. The potential significance of this observation should be explored in future investigations. Moreover, the prevalence of SET overexpression in the subgroup of patients with methacronous metastases was 16.1%, which is markedly lower than the prevalence of 28.1% previously reported in CRC patients with synchronous metastases at diagnosis [12].

The fact that SET overexpression only associates with relapse in those cases with stage-II disease could be due to the fact that stage III encompasses patients with more advanced disease, where the presence of this alteration could be a less relevant event in the effort to determine relapse than in those cases with stage-II CRC. However, the number of cases with stage II and progression included in our study is still very low and represents a relevant limitation. Therefore, further validation of our observations in forthcoming studies is required to confirm the clinical impact of SET predicting relapse in CRC sage II. In addition, overexpression of SET was significantly associated with shorter time to metastasis and with time to progression from metastatic diagnosis in the subgroup of patients who develop distant relapse (Figure 4), further suggesting that this alteration indicates more aggressive disease. Our results also show that SET overexpression predicts shorter overall survival and that the clinical impact on prognosis is especially significant in the subgroup of patients older than 70 years of age. Despite the fact that CRC is a disease of the elderly, with a median age of over 70 years, gradual improvements in therapy have mainly impacted the subgroup of younger patients, likely due to both patient and disease-specific factors. It would therefore be very relevant to identify novel alterations that can aid in predicting prognosis in this subgroup of CRC patients and can be used to develop novel targeted therapies. Our results suggest that SET overexpression predicts poor outcome in elderly CRC patients and that this overexpression could also be predictive of a clinical benefit derived from the use of SET targeting therapies such as the PP2A activator FTY720 or specific SET antagonists [30,31,32,33,34].

As with previous observations in metastatic CRC, which evidenced how miR-199b was downregulated in around 50% of SET-overexpressing cases, here we observed that miR-199b downregulation was significantly associated with SET overexpression and that 4 out of 7 CRC patients with SET overexpression showed reduced miR-199b levels (Table S4). Moreover, total miR-199b expression levels were inversely correlated with SET in our cohort (Figure S6), a finding which further strengthens the case for the role played by miR-199b in regulating SET in early-stage CRC patients. These results may also indicate that other molecular alterations contribute to the deregulation of SET in those cases with SET OE and normal miR-199b expression, though this issue will be investigated in forthcoming papers.

Interestingly, our findings demonstrate a key role of SET in cancer progression mediating cell migration, colony-formation and EMT but these observations for SET in CRC have to be also evaluated in other tumor models with SET deregulation. Moreover, these findings are in concordance with the previously reported role of SET in migration [15], but the potential role of Rac-1 in this case remains to be explored in future studies. Furthermore, we show here the potential impact of SET as a novel molecular target in CRC. In this regard, the use of SET antagonists such as OP449 and FTY720 have emerged in the last years as novel therapeutic approaches in human tumors with SET overexpressed and could also be useful in early-stage CRC patients. OP449 is a specific, cell-penetrating peptide against SET, which has shown anticancer properties in leukemia, prostate, breast and gastric cancers [18,35,42,43], but its potential usefulness in CRC remains to be evaluated. FTY720 is an immunosuppressant currently used in the sclerosis multiple, which has demonstrated potent antitumor effects in many cancers, including CRC [44].

## 5. Conclusions

In conclusion, we show that SET overexpression is a common alteration that contributes to CRC progression, thereby enhancing both cell migration and anchorage-independent cell growth of CRC cells. Moreover, SET overexpression predicts poor outcome, and its prognostic value is particularly significant in patients older than 70 years of age. We have further identified the key function likely played by miR-199b downregulation as a molecular mechanism involved in SET deregulation in many of the SET-overexpressing cases. Our results indicate that SET could be used to anticipate undesirable relapses in stage-II CRC patients and highlight the potential impact of SET inhibition as a novel therapeutic strategy in CRC, which needs to be fully clarified in forthcoming studies.

## Figures and Tables

**Table 1:** Association between SET and clinical and molecular parameters in 247 CRC patients without metastatic disease at diagnosis.

	No. Cases	No. SET Low (%)		No. SET High (%)		*p*
SET	247	209 (84.6)		38 (15.4)		
Sex	247	209		38		0.674
Male	155	130	(83.9)	25	(16.1)	
Female	92	79	(85.9)	13	(14.1)	
Age	236	199		37		0.349
<70	86	70	(81.4)	16	(18.6)	
>70	150	129	(86)	21	(14)	
ECOG	223	190		33		**0.021**
0–1	205	178	(86.8)	27	(13.2)	
2–3	18	12	(66.7)	6	(33.3)	
T	247	209		38		0.824
1	7	6	(85.7)	1	(14.3)	
2	48	43	(89.6)	5	(10.4)	
3	155	128	(82.6)	27	(17.4)	
4	29	25	(86.2)	4	(13.8)	
x	8	7	(87.5)	1	(12.5)	
N	247	209		38		0.421
0	117	102	(87.2)	15	(12.8)	
1	67	56	(83.6)	11	(16.4)	
2	44	34	(77.3)	10	(22.7)	
x	19	17	(89.5)	2	(10.5)	
Stage	247	208		38		0.302
I–II	117	102	(87.2)	15	(12.8)	
III	130	107	(82.3)	23	(17.7)	
Site of primary tumor	241	203		38		0.699
Colon	165	140	(84.8)	25	(15.2)	
Rectum	76	63	(82.9)	13	(17.1)	
Progression (local or distant)	247	209		38		0.144
No	156	136	(87.2)	20	(12.8)	
Yes	91	73	(80.2)	18	(19.8)	

**Table 2:** Association between SET and progression (local or distant) in 207 stages II-III CRC patients.

**Stage II**	**No. Cases**	**No. SET Low (%)**		**No. SET High (%)**		***p***
Progression	77	66		11		**0.008**
No	59	54	(81.8)	5	(45.5)	
Yes	18	12	(18.2)	6	(54.5)	
**Stage III**	**No. Cases**	**No. SET low (%)**		**No. SET high (%)**		***p***
Progression	130	107		23		0.882
No	64	53	(49.5)	11	(47.8)	
Yes	66	54	(50.5)	12	(52.2)	

**Table 3:** Univariate and multivariate Cox analyses in the cohort of 149 patients with early-stage CRC.

		Univariate OS Analysis				Multivariate OS Cox Analysis			
		HR	95% CI		*p*	HR	95% CI		*p*
			Lower	Upper			Lower	Upper	
Age					0.319				-
	<70	1.000							
	>70	1.352	0.747 to 2.445			-	-		
Stage					**0.003**				0.235
	II	1.000				1.000			
	III	3.421	1.540 to 7.601			1.956	0.646 to 5.926		
ECOG					**<0.001**				**<0.001**
	0–1	1.000				1.000			
	2–3	2.797	1.975 to 3.961			2.653	1.845 to 3.814		
T					**0.002**				0.057
	1–2	1.000				1.000			
	>2	1.785	1.226 to 2.599			1.488	0.989 to 2.240		
N					**0.001**				0.275
	0–1	1.000				1.000			
	2–x	1.599	1.223 to 2.091			1.263	0.830 to 1.921		
SET					**0.001**				**0.010**
	No	1.000				1.000			
	Yes	2.873	1.547 to 5.335			2.387	1.229 to 4.634		

OS: overall survival; CI: confidence interval, and HR: hazard-ratio. Bold: *p* < 0.01.

## Supplementary Materials

The following are available online at https://www.mdpi.com/2077-0383/8/3/346/s1, Figure S1: SET modulates cell migration in CRC cells, Figure S2: Time-course analysis of a wound healing assay in SW480 cells with SET silenced and with or without ectopic SET expression, Figure S3: Time-course analysis of a wound healing assay in HT-29 cells with SET silenced and with or without ectopic SET expression, Figure S4: Ectopic SET expression restores transwell migration in CRC cells with SET silenced, Figure S5: Clinical impact of SET in time to progression from metastatic diagnosis and in the cohort stratified by age, Figure S6: Box-plot showing miR-199b expression levels in patients with (*n* = 7) and without (*n* = 22) SET overexpression, Table S1: Clinical and molecular characteristics of a series of 231 CRC patients without metastatic disease at diagnosis, Table S2: Association of ECOG status with age and stage parameters, Table S3: Univariate and multivariate Cox analyses in the subgroup of elderly patients with early-stage CRC (*n* = 81), Table S4: Statistical correlation between SET overexpression and miR-199b downregulation in 29 early-stage CRC.
