# Supplementary material for: Deregulation of SET is Associated with Tumor Progression and Predicts Adverse Outcome in Patients with Early-Stage Colorectal Cancer
Source: J Clin Med. 2019 Mar 12;8(3):346. doi: 10.3390/jcm8030346 (PMC6463201; doi:10.3390/jcm8030346)
Supplement: Supplementary file 1 [file jcm-08-00346-s001.pdf]

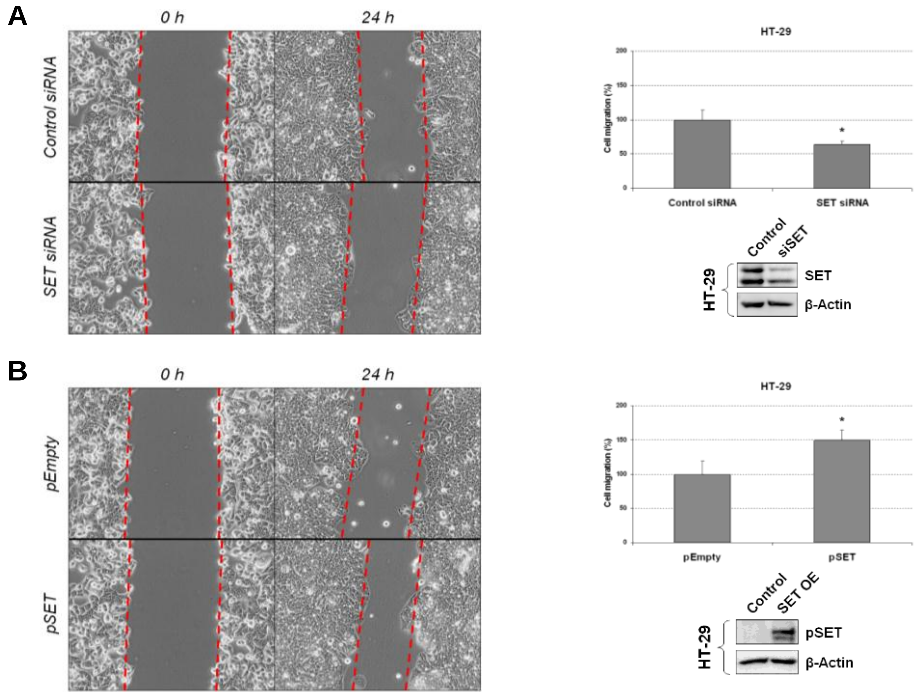

Supplementary Figure 1

Figure S1. SET modulates cell migration in CRC cells. Wound- healing assay showing migration in HT-29 cells after ectopic (**A**) SET silencing or (**B**) SET overexpression. Dashed lines represent the migration border; OE: overexpression; *P < 0.05; **P < 0.01.

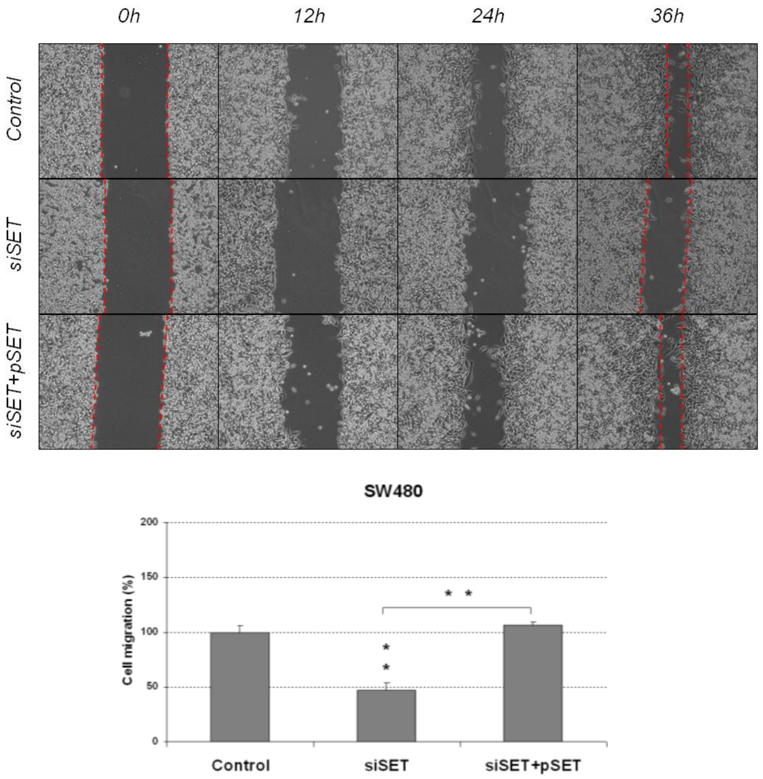

Supplementary Figure 2

Figure S2. Time-course analysis of a wound healing assay in SW480 cells with SET silenced and with or without ectopic SET expression. Dashed lines represent the migration border; OE: overexpression; *P < 0.05; **P < 0.01.

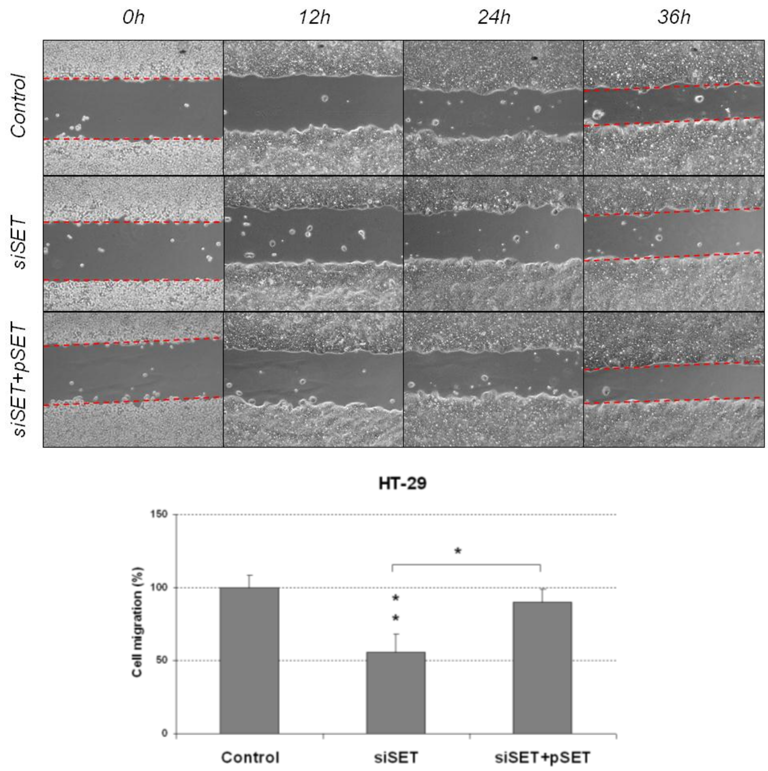

Supplementary Figure 3

Figure S3. Time-course analysis of a wound healing assay in HT-29 cells with SET silenced and with or without ectopic SET expression. Dashed lines represent the migration border; OE: overexpression; $^*P < 0.05$; $^{**}P < 0.01$.

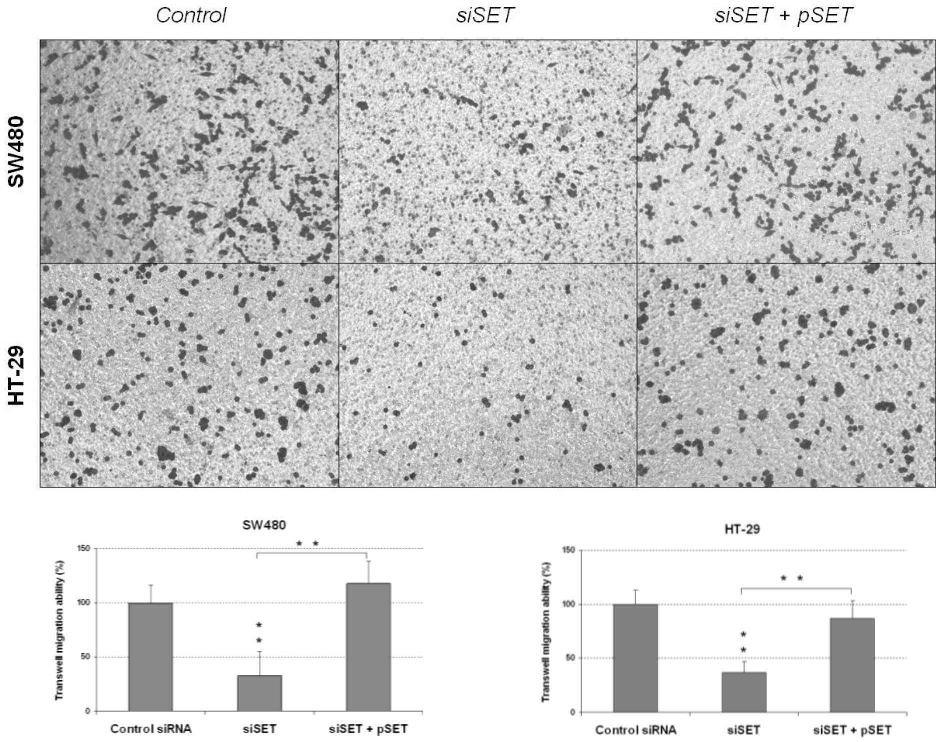

Supplementary Figure 4

Figure S4. Ectopic SET expression restores transwell migration in CRC cells with SET silenced. Transwell migration assay in SW480 and HT-29 cells after SET silencing with or without SET overexpression; * $P < 0.05$; ** $P < 0.01$.

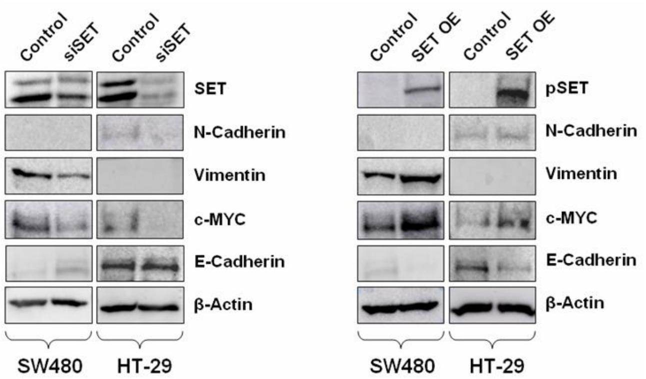

Supplementary Figure 5

Figure S5. SET modulates the expression of c-MYC and EMT proteins.

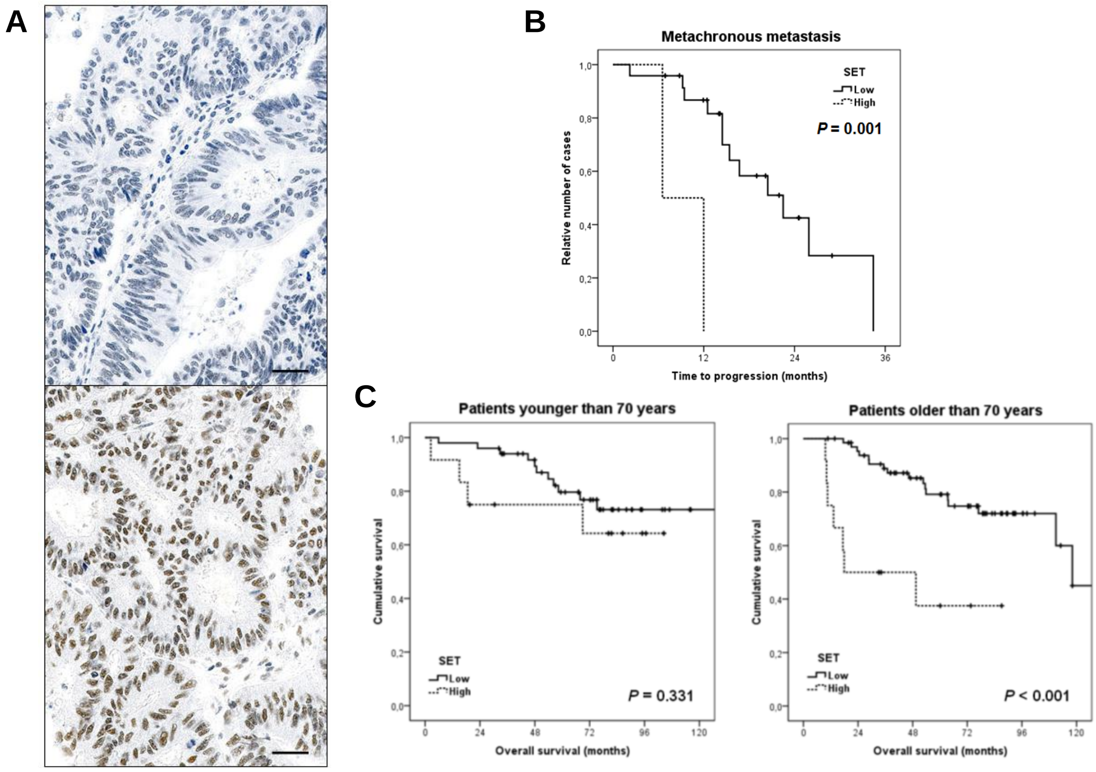

Supplementary Figure 6

Figure S6. (**A**) Immunohistochemical detection of SET expression in patients with early-stage CRC showing SET-negative and -positive stainings. The line shows 25 µm. Original magnification x400. (**B**) Time to progression from metastatic diagnosis in the subgroup of patients with metachronous metastases (**C**) Kaplan-Meier analyses of OS in the subgroups of patients younger (N = 62) and older than 70 years (N = 79).

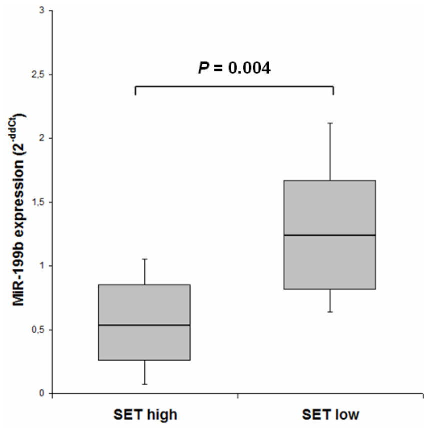

Supplementary Figure 7

Figure S7. Box-plot showing miR-199b expression levels in patients with (*N* = 7) and without (*N* = 22) SET overexpression.

Table S1. Clinical and molecular characteristics of a series of 231 CRC patients without metastatic disease at diagnosis.

		No. (%)	
Sex			
	Male	155	(62.8)
	Female	92	(37.2)
Age			
	<70	86	(36.4)
	≥70	150	(62.8)
ECOG			
	0-1	205	(91.9)
	2-3	18	(8.1)
T			
	1	7	(2.8)
	2	48	(19.4)
	3	155	(62.7)
	4	29	(11.8)
	x	8	(3.3)
N			
	0	117	(47.4)
	1	67	(27.1)
	2	44	(17.8)
	x	19	(7.7)
Stage			
	I	39	(15.8)
	II	78	(31.6)
	III	130	(52.6)
Site of primary tumor			
	Colon	165	(68.5)
	Rectum	76	(31.5)
Progression (local or distant)			
	No	156	(63.2)
	Yes	91	(36.8)

Table S2. Association of ECOG status with age and stage parameters.

ECOG	No. Cases	No. ECOG 0-1 (%)	No. ECOG 2-3 (%)	*p*
Age	223	205	18	0.060
<70	83	80 (81.9)	3 (18.1)	
>70	140	125 (87.1)	15 (12.9)	
Stage	223	205	18	**0.014**
I-II	99	96 (89)	3 (11)	
III	124	109 (82.3)	15 (17.7)	

Table S3. Univariate and multivariate Cox analyses in the subgroup of elderly patients with early-stage CRC (*N*= 81).

	Univariate OS analysis				Multivariate OS Cox analysis			
		95% CI				95% CI		
	HR	Lower	Upper	Significance	HR	Lower	Upper	Significance
Stage				**0.041**				0.173
II	1.000				1.000			
III	3.068	1.046 to 8.998			2.158	0.714 to 6.524		
ECOG				**0.001**				**0.004**
0-1	1.000				1.000			
2-3	2.477	1.474 to 4.162			2.223	1.294 to 3.819		
T				0.470				-
1-2	1.000							
>2	1.292	0.644 to 2.593			-	-		
N				0.165				-
0-1	1.000							
2-x	1.307	0.896 to 1.908			-	-		
SET				**0.001**				**0.007**
No	1.000				1.000			
Yes	4.924	1.984 to 12.223			3.677	1.438 to 9.400		

OS: overall survival; CI: confidence interval, HR: hazard ratio.

Table S4. Clinical and molecular characteristics of the subgroup of 29 cases with both SET and miR-199b data.

		No. (%)	
Sex			
	Male	21	(72.4)
	Female	8	(27.6)
Age			
	<70	10	(38.5)
	≥70	16	(61.5)
T			
	1	0	(0)
	2	6	(20.7)
	3	17	(58.6)
	4	5	(17.2)
	x	1	(3.5)
N			
	0	10	(34.5)
	1	11	(37.9)
	2	7	(24.1)
	x	1	(3.5)
Stage			
	I-II	10	(34.4)
	III	19	(65.6)
Site of primary tumor			
	Colon	20	(69)
	Rectum	9	(31)
SET			
	Low	22	(75.9)
	High	7	(24.1)

Table S5. Statistical correlation between SET overexpression and miR-199b downregulation in 29 early-stage CRC.

		SET				
	**Total**	**Low**	**(%)**	**High**	**(%)**	***p***
MiR-199b	29	22		7		**0.010**
Low	4	1	(25)	3	(75)	
High	25	21	(84)	4	(16)	
